# Measuring breakfast dietary patterns in Spanish youth: Reliability of the Spanish Youth Breakfast Consumption Questionnaire (SYBC-Q)

**DOI:** 10.1177/02601060251368960

**Published:** 2025-09-01

**Authors:** Pablo Campos-Garzón, Romina Gisele Saucedo-Araujo, Manuel Herrador-Colmenero, Yaira Barranco-Ruiz

**Affiliations:** 1 Faculty of Health Sciences, 4512 University of Lethbridge, Lethbridge, AB, Canada; 2 Department of Global Public Health, Karolinska Institutet, Stockholm, Sweden; 3 Department of Physical Education and Sports, Faculty of Education and Sport Sciences, Sport and Health University Research Institute (iMUDS), 16741 University of Granada, Melilla, Spain; 4 La Inmaculada Teacher Training Centre, Sport and Health University Research Institute (iMUDS), 16741 University of Granada, Granada, Spain; 5 Department of Physical Education and Sports, Faculty of Sport Sciences, Sport and Health University Research Institute (iMUDS), 16741 University of Granada, Granada, Spain

**Keywords:** Test–retest, youth, dietary patterns, breakfast composition, breakfast quality

## Abstract

**Background:** Reliable tools to assess breakfast habits are essential, as breakfast quality is linked to health and development in youth. However, validated instruments for Spanish children and adolescents are scarce. **Objectives:** To assess the reliability of the Spanish Youth Breakfast Consumption Questionnaire (SYBC-Q) in a sample of Spanish children and adolescents. **Methods:** A total of 248 young people participated in the study: 77 children (46.8% girls; 10.7 ± 0.7 years) and 171 adolescents (50.3% girls; 14.1 ± 1.7 years). They completed the questionnaire twice, 14 days apart, on the same days of the week and schedule. The tool includes 21 items grouped into seven food categories and two breakfast quality scores. Test-retest reliability was analyzed using the kappa coefficient (κ) and Spearman's correlation (Rho). Analyses were performed separately by age group and gender. **Results:** Children showed an acceptable to good level of agreement and a moderate to high correlation between test and retest (κ: 0.23–0.71; Rho: 0.37–0.76). However, low agreement and poor correlation were found for ‘Take Cheese’ and ‘Take Milk dessert’ in girls (both, κ < 0.20; Rho < 0.30). Adolescents presented an acceptable to perfect level of agreement and a low to high correlation between test and retest (κ: 0.24–0.82; Rho: 0.24–0.82), but a low level of agreement in the items ‘Take pâte’ in girls and ‘Take dried fruits’ in boys (both, κ: < 0.20; Rho: < 0.30). **Conclusion:** Overall, the SYBC-Q is a reliable tool for assessing the breakfast consumption and quality in Spanish children and adolescents.

## Introduction

Breakfast is commonly considered the most important meal of the day (O’Neil et al., 2014) and an essential part of a healthy diet (Arenaza et al., 2018; Giménez-Legarre et al., 2020). This view is supported by numerous benefits associated with breakfast, including positive effects on weight control and psychological and psychosocial health (Betts et al., 2016; Spence, 2017). Given the exponential growth of overweight and obesity in the young population (Abarca-Gómez et al., 2017), one of the most studied topics related to breakfast has been its impact on body composition in children and adolescents (Monzani et al., 2019). Children who consume bread, sugar and/or sugary drinks in the breakfast instead of fruit or vegetables are more likely to be obese (Kyriazis et al., 2012), as well as eating high-sugar cereals increases total daily sugar intake and reduces breakfast quality (Harris et al., 2011). Therefore, as Al-Hazzaa et al. (2020) pointed out, it is not just a question of whether or not to have breakfast in children, but what they eat.

Breakfast patterns vary widely across cultures. In Spain, the most common breakfast for young people is a dairy product plus bread or cereal (Cuadrado-Soto et al., 2020). In France, it typically includes whole-grain cereal, fruit, and dairy (Bellisle et al., 2018). In Mexico, many children eat corn tortillas (Afeiche et al., 2017), in the USA, they often choose ready-to-eat cereals (Albertson et al., 2003), and in Saudi Arabia, cheese sandwiches or fried eggs are common (Al-Hazzaa et al., 2020). Because of these cultural differences, country-specific guidelines on breakfast composition are necessary for accurate quality assessment (Gibney et al., 2018). Spain, for example, has issued recommendations for a healthy breakfast for children and adolescents (Serra-Majen et al., 2000). However, despite these well-established benefits, there remains a significant gap in reliable tools to assess breakfast quality (Moreno Aznar et al., 2021) the role of breakfast in the health of Spanish children and adolescents is critical, and there is a need for tools that allow for adequate evaluation of its quality. Moreover, given the differences between countries and cultures, there is a need for culturally adapted tools which can measured the daily breakfast patterns in young population (Martinez et al., 2024).

Although breakfast quality has traditionally been assessed with food diaries (Caon et al., 2022; Kawalec and Pawlas, 2021), recent work favours short food questionnaires (SFQs) (Golley et al., 2017), because they are quick to administer, can be self-completed, impose less respondent burden, and are easy to analyse (Magarey et al., 2011). However, most SFQs in young people still record only whether breakfast is eaten, not its composition (Flood et al., 2014; Guldan et al., 2014). Golley et al. (2017) highlighted substantial gaps in the reliability and validity of such tools, and Park et al. (2023) underlined the need for instruments that can track breakfast-quality changes over time in early childhood. Given the importance of breakfast composition and its associated benefits, it is necessary to study which foods make up the breakfast.

To date, no study has examined the reliability of an SFQ aligned with recommendations from the Spanish Diabetes Foundation and the Spanish Society of Community Nutrition in Spanish youth. Such tools can help establish the basis for assessing breakfast quality in youth and promoting healthy breakfast habits into adulthood (Appleton et al., 2018). Therefore, the current study aimed to assess the reliability (i.e., constancy, coherence, and consistency) of a SFQ: the Spanish Youth Breakfast Consumption Questionnaire (SYBC-Q) in a sample of Spanish children and adolescents.

## Methods

### Design

This is a test–retest study conducted between March and April 2018, in which participants completed the SYBC-Q twice, 14 days apart. According to the scientific literature, a two-week interval between test and retest is sufficient to minimize the influence of participants’ initial responses on the retest (Štefan et al., 2017). This test–retest study is part of the previous phase of the PACO study (Pedalea y Anda al Colegio/Cycle and Walk to School). In addition, the present study has been written in accordance with The Strengthening the Reporting of Observational Studies (STROBE) Statement: guidelines for reporting observational studies (see supplementary Table S1).

### Participants

A total sample of 285 students, including 85 children (5–12 years old) and 200 adolescents (12–18 years old) from Granada (Spain), were invited to participate in this study. To be included in the analysis, participants had to provide valid test and retest SYBC-Q data (i.e., answer at least one item from the SYBC-Q in both the test and retest). A total of 8 children and 29 adolescents did not provide valid data; therefore, the final sample for the current study consisted of 248 participants (171 adolescents).

### Procedures

One public primary school and one public secondary school from the city of Alhendín (Granada, Spain) were selected through convenience sampling. These schools had previously participated in research projects conducted by the study team, which facilitated access and collaboration. Initially, researchers met with the school principal and the Physical Education teaching staff, as well as with the students, to provide detailed information about the PACO project, which aimed to promote active commuting to and from school. However, the specific purpose of the SYBC-Q questionnaire was not disclosed to the participants in order to minimize response bias and ensure that breakfast-related responses reflected their usual habits. Once the schools agreed to participate, parents or legal guardians who also consented to their children's involvement were required to sign an informed consent form. Participants completed the SYBC-Q twice (test and retest), with a 14-day interval between administrations. Both sessions were conducted under fully standardized conditions, including the same weekday (Tuesday or Thursday), within the same time window (during Physical Education lessons), and under the supervision of the same four researchers. Thus, a standardized administration protocol was followed, which included: (a) use of the same classroom setting; (b) identical oral instructions read aloud by the researchers; (c) a quiet, distraction-free environment; and (d) individual and silent questionnaire completion without peer interaction. Personal data, such as gender and age, were also self-reported by participants using a paper-based questionnaire. The Fujitsu fi-7160 scanner and the Data-scan software version 5.7.7 were used to digitize the questionnaires and create the database.

### Spanish youth breakfast consumption questionnaire

The SYBC-Q aimed to determine which kind of food usually selects children and adolescents for their breakfast and evaluate its quality. To evaluate the breakfast composition and the breakfast quality, participants were instructed to complete this short questionnaire in the item number 33 of the PACO Study Questionnaire (http://profith.ugr.es/pages/investigacion/recursos/cuestionario-alumnos-v4).

The SYBC-Q begins with the following question: ‘What do you usually have for breakfast on weekdays before going to school? (You can choose more than one option)’, followed by a list of 21 items representing typical foods in Spanish breakfasts. The response option for all items was either ‘yes’ or ‘no’, indicating whether participants usually consume these foods. These 21 foods represent the seven essential food groups, according to the guidelines of the Spanish Diabetes Foundation (https://www.sediabetes.org/), the Spanish Society of Community Nutrition (https://www.sennutricion.org/), and the EnKid Study (Serra-Majen et al., 2000). Additionally, the 21 foods were classified as healthy or unhealthy based on expert judgment. The foods included in the SYBC-Q are listed in Table 1. The Spanish version of the questionnaire is provided in Supplementary Table S2, and an English translation is available in Table S3 for reference purposes only.

**Table 1.** Items and food categories in the SYBC-Q.

Food groups		Healthy/ non-healthy
*Group 1: Dairy group*		
1	Milk	H
2	Natural yogurt	H
3	Cheese	H
4	Milk dessert	NH
*Group 2: Meat and protein group*		
5	Ham	H
6	Eggs	H
7	Pâté, fatty cold cuts, sausages, etc.	NH
*Group 3: Dried fruits group*		
8	Dried fruits (walnuts, cashews, pistachios, almond, etc.)	H
*Group 4: Vegetables group*		
9	Vegetables such as tomato, carrot, avocado, …	H
*Group 5: Fruit group*		
10	Piece of natural fruit	H
11	Non-natural fruit (fruit in syrup, packed fruit juice mix)	NH
*Group 6: Cereal and sugar group*		
12	White bread	H
13	Wheat bread	H
14	Non-sugar cereal such as oat	H
15	Cereals with sugar	NH
16	Sugar (more than one teaspoon)	NH
17	Honey (up to one teaspoon)	H
18	Cacao or chocolate without sugar	H
19	Factory baked goods	NH
*Group 7: Oil and fats group*		
20	Olive oil	H
21	Butter	NH

Note: H = healthy; NH = non-healthy or unhealthy.

The SYBC-Q uses two complementary methods to assess breakfast quality, focusing on the balance of its nutritional composition, the healthiness of the included foods, and the number of unprocessed, healthy items. According to the EnKid Study (Serra-Majen et al., 2000), the main food groups that should be included in a healthy and balanced breakfast are Groups 1, 5, 6 and 7 (see Table 1). For this reason, the foods consumed by participants are first identified and categorized by food groups to analyze which ones are commonly included or omitted. Based on the presence or absence of the main food groups, breakfast is classified into five categories: (a) very high-quality full breakfast (contains foods from the four main groups and additionally from the other groups); (b) high-quality full breakfast (contains foods from at least the four main groups); (c) good quality breakfast (contains at least foods from the dairy groups, cereals, and fruit); (d) breakfast of improvable quality (it lacks foods from one of the dairy, cereal and fruit groups); and (e) insufficient quality breakfast (it lacks foods from two of the dairy, cereal, and fruit groups). In addition, to evaluate the healthy composition of breakfast, the healthy unprocessed foods usually consumed in the breakfast were also quantified. The healthiest breakfast was considered when four or more healthy foods were included in the breakfast (i.e., irrespective of the food group they come from). The categories according to the number of healthy unprocessed food consumed were the following: (a) highly healthy (4 or >4 healthy unprocessed foods usually consumed); (b) Healthy (3 healthy unprocessed foods usually consumed); (c) Moderately healthy (2 healthy unprocessed foods usually consumed); and (d) Unhealthy (0 or only 1 healthy unprocessed food usually consumed).

### Statistical analyses

A descriptive statistic was performed with as frequencies and percentages for categorical variables and means and standard deviations for the continuous variables. The test-retest reliability was calculated using the kappa coefficient and the Spearman correlation coefficient (Rho) for the seven food groups (i.e., dairy, meat and protein, dried fruits, fruit, cereal and sugar, and oils and fats) and the foods that compose them. Moreover, the same analyses were performed to examine the level of agreement and correlation for the proposed scores (i.e., presence or absence of food groups score and healthy unprocessed food score). The analyses were carried out separately by age (i.e., children and adolescents) and by gender (i.e., girls and boys). Moreover, to classify the results obtained from the weighted kappa, cut points proposed by Landis and Koch (1977) were used: 0.00–0.20 = poor agreement; 0.21–0.40 = acceptable agreement; 0.41–0.60 = moderate agreement; 0.61–0.80 = substantial/good; 0.81–1.00 = almost perfect/very good agreement. The Spearman correlation coefficients were interpreted as low ( < 0.30), moderate (0.30–0.50), and high ( > 0.50) (Segura-Díaz et al., 2021). All the analyses were performed with SPSS for Windows version 23 (SPSS Inc., Chicago, IL, USA), establishing a level of statistical significance of *p < *0.05.

## Results

### Descriptive characteristics of the participants

A total of 248 young people participated in the study, of whom 77 were children (46.8% girls) aged 10.7 ± 0.7 years old, and 171 adolescents (50.3% girls) aged 14.1 ± 1.7 years old. Moreover, the number of participants and the percentages of different foods included in their breakfast are presented in the supplementary material (Tables S4 and S5).

### Reliability

Kappa coefficient and Rho for the test-retest reliability of the SYBC-Q in children and by gender are shown in Table 2. The level of agreement between the test and retest of the SYBC-Q showed to be acceptable to good for all the foods and food groups (kappa ranged from 0.24 to 0.73; all, *p < *0.05), except for the food ‘Take cheese’, which showed a poor level of agreement (kappa = 0.06; *p = *0.06). The correlation between the test and retest of the SYBC-Q was moderate to high for most foods and food groups (Rho ranged from 0.31 to 0.71; all, *p < *0.05), except for the item ‘Take cheese’, which showed a low correlation (Rho = 0.06; *p = *0.63). In the analysis by gender, boys showed for all SYBC-Q foods and food groups an acceptable to good agreement (kappa ranged from 0.35 to 0.79) and a moderate to good association (Rho ranged from 0.35 to 0.88; all, *p < *0.05) between test and retest, except for the food ‘Take cheese’ which showed a low agreement (kappa = ‒0.06; *p = *0.67) and a low correlation (Rho = ‒0.07; *p = *0.59). Regarding the girls’ group, they showed an acceptable to perfect agreement for all foods and food groups (kappa ranged from 0.30 to 1.00; all, *p < *0.05) and the correlation between test and retest of the SYBC-Q was moderate to high (Rho ranged from 0.33 to 1.00; all, *p < *0.05). Nevertheless, the level of agreement and correlation for the foods ‘Take cheese’ and ‘Take milk dessert’ were poor (kappa = 0.11; *p = *0.40; and kappa = 0.23; *p = *0.16, respectively) and low (Rho = 0.11; *p = *0.50; and Rho = 0.23; *p = *0.17, respectively), and the level of agreement for the food groups ‘Dairy group’ was poor (kappa = 0.12; *p = *0.32). Regarding the scores, the Presence or absence of food groups score and the Healthy unprocessed food score showed a moderate to good level of agreement (kappa ranged from 0.56 to 0.79 and from 0.56 to 0.62, respectively; all, *p < *0.05) in children and by gender and a high correlation (Rho ranged from 0.62 to 0.84 and from 0.64 to 0.72, respectively; all, *p < *0.05) between test and retest of the SYBC-Q.

**Table 2:** Test–retest reliability of the SYBC-Q among children by gender.

	Children								
	All			Boys			Girls		
Food groups/foods	*n*	Kappa	Rho	*n*	Kappa	Rho	*n*	Kappa	Rho
*Dairy group*	75	0.24	0.39	39	0.35	0.44	36	0.12^a^	0.33
Take milk	77	0.36	0.37	41	0.36	0.37	36	0.36	0.37
Take natural yogurt	76	0.58	0.58	40	0.44	0.44	36	0.68	0.68
Take cheese	76	0.06^a^	0.06^a^	40	−0.06^a^	−0.07^a^	36	0.11^a^	0.11^a^
Take milk dessert	75	0.31	0.31	39	0.39	0.41	36	0.23^a^	0.23^a^
*Meat and protein group*	73	0.57	0.76	37	0.66	0.88	35	0.45	0.61
Take ham	74	0.73	0.74	38	0.79	0.79	36	0.67	0.71
Take eggs	73	0.41	0.41	38	0.53	0.54	35	1.00	1.00
Take pâté	74	0.54	0.55	39	0.54	0.56	35	0.53	0.53
*Dried fruits group*	72	0.71	0.71	37	0.62	0.64	35	0.78	0.79
Take dried fruits	72	0.71	0.71	37	0.62	0.64	35	0.78	0.79
*Vegetables group*	75	0.58	0.58	39	0.69	0.69	36	0.47	0.48
Take vegetables	75	0.58	0.58	39	0.69	0.69	36	0.47	0.48
*Fruit group*	75	0.63	0.67	40	0.55	0.57	36	0.69	0.81
*Take piece of natural fruit*	76	0.64	0.63	40	0.48	0.49	36	0.80	0.82
*Take natural fruit juice*	75	0.64	0.64	40	0.51	0.51	35	0.77	0.78
*Cereal and sugar group*	72	0.23	0.66	37	0.23	0.70	34	0.23	0.63
*Take white bread*	74	0.51	0.47	40	0.44	0.49	34	0.48	0.50
*Take wheat bread*	75	0.55	0.55	40	0.49	0.52	35	0.55	0.56
*Take cereal oat*	72	0.65	0.65	37	0.44	0.46	35	0.80	0.82
*Take cereals with sugar*	75	0.53	0.53	40	0.55	0.55	35	0.49	0.50
*Take sugar*	75	0.53	0.53	40	0.66	0.67	35	0.28	0.30
*Take honey*	75	0.67	0.67	39	0.69	0.70	36	0.65	0.66
*Take cacao*	76	0.71	0.71	40	0.75	0.76	36	0.68	0.68
*Take factory baked goods*	75	0.61	0.62	39	0.58	0.60	36	0.64	0.64
*Oil and fats group*	74	0.44	0.63	38	0.41	0.65	36	0.47	0.60
*Take olive oil*	74	0.70	0.70	38	0.68	0.68	36	0.72	0.72
*Take butter*	74	0.54	0.54	38	0.55	0.55	36	0.53	0.54
*Scores*									
Presence or absence of food groups score	72	0.67	0.72	37	0.79	0.84	35	0.56	0.62
Healthy unprocessed food score	72	0.59	0.69	37	0.56	0.64	35	0.62	0.75

Note: *n*:  sample size; Rho: The Spearman correlation coefficient.

^a^ Not statistically significant agreement (Kappa) and/or not statistically significant correlation (Spearman correlation) (*p* > 0.05).

Test‒retest reliability of the SYBC-Q among adolescents and by gender is presented in Table 3. The results indicated an acceptable to perfect level of agreement (kappa ranged from 0.24 to 0.82; all, *p < *0.05) and a moderate to high correlation (Rho ranged from 0.24 to 0.82; all, *p < *0.05) between foods and food groups of the SYBC-Q in the test and retest. As for the analysis by gender, boys showed an acceptable to good level of agreement (kappa ranged from 0.31 to 0.76; all, *p < *0.05) and a moderate to high correlation (Rho ranged from 0.24 to 0.82, all, *p < *0.05) in all the food and foods group, except for the food ‘Take dried fruits’, which showed a poor agreement (kappa = 0.18; *p = *0.07) and low correlation (Rho = 0.20; *p = *0.07). Regarding girls, they showed an acceptable to perfect level of agreement (kappa ranged from 0.22 to 1.00; all, *p < *0.05) and a moderate to high correlation in most foods and food groups (Rho ranged from 0.26 to 1.00; all, *p < *0.05), except in the food ‘Take pâté’ (kappa = 0.18; *p = *0.09; Rho = 0.18; *p = *0.09). Regarding the scores, there was a moderate level of agreement observed in the ‘Presence or absence of food groups score’ (kappa ranged from 0.42 to 0.60; all, *p < *0.05) and a moderate to high correlation was found in adolescents and by gender (Rho ranged from 0.48 to 0.69; all, *p < *0.05) between test and retest of the SYBC-Q. Moreover, the Healthy unprocessed foods score showed a moderate to good level of agreement (kappa ranged from 0.48 to 0.67; all, *p < *0.05) and a high correlation (Rho ranged from 0.72 to 0.88; all, *p < *0.05) in adolescents and by gender between test and retests of the SYBC-Q.

**Table 3:** Test–retest reliability of the SYBC-Q among adolescents by gender.

	Adolescents								
	All			Boys			Girls		
Food groups/foods	*n*	Kappa	Rho	*n*	Kappa	Rho	*n*	Kappa	Rho
*Dairy group*	167	0.53	0.67	82	0.46	0.63	85	0.54	0.69
Take milk	170	0.82	0.82	84	0.75	0.75	86	0.86	0.86
Take natural yogurt	170	0.57	0.57	84	0.46	0.46	86	0.61	0.62
Take cheese	167	0.47	0.48	82	0.32	0.34	85	0.65	0.65
Take milk dessert	170	0.61	0.61	84	0.67	0.68	86	0.55	0.55
*Meat and protein group*	167	0.50	0.65	82	0.50	0.69	85	0.51	0.61
Take ham	170	0.58	0.58	85	0.63	0.63	85	0.54	0.54
Take eggs	167	0.58	0.61	82	0.39	0.43	85	1.00	1.00
Take pâté	171	0.27	0.27	85	0.31	0.31	86	0.18^a^	0.18^a^
*Dried fruits group*	171	0.24	0.24	85	0.18^a^	0.20^a^	86	0.26	0.26
Take dried fruits	171	0.24	0.24	85	0.18^a^	0.20^a^	86	0.26	0.26
*Vegetables group*	170	0.62	0.62	84	0.71	0.72	86	0.51	0.51
Take vegetables	170	0.62	0.62	84	0.71	0.72	86	0.51	0.51
*Fruit group*	169	0.47	0.57	83	0.48	0.66	86	0.46	0.50
Take piece of natural fruit	169	0.57	0.57	83	0.62	0.63	86	0.53	0.53
Take natural fruit juice	169	0.53	0.53	83	0.56	0.56	86	0.50	0.51
*Cereal and sugar group*	164	0.29	0.76	80	0.35	0.77	84	0.22	0.74
Take white bread	171	0.61	0.61	85	0.75	0.76	86	0.48	0.48
Take wheat bread	171	0.56	0.56	85	0.57	0.58	86	0.54	0.55
Take cereal oat	169	0.49	0.49	84	0.32	0.32	85	0.59	0.59
Take cereal with sugar	171	0.49	0.49	85	0.42	0.42	86	0.55	0.57
Take sugar	169	0.56	0.56	84	0.48	0.48	85	0.63	0.64
Take honey	164	0.61	0.61	80	0.61	0.61	84	0.59	0.59
Take cacao	169	0.69	0.70	83	0.76	0.77	86	0.63	0.64
Take factory baked goods	169	0.60	0.60	84	0.62	0.63	85	0.57	0.57
*Oil and fats group*	168	0.59	0.70	84	0.61	0.77	84	0.56	0.64
Take olive oil	168	0.59	0.59	84	0.59	0.59	84	0.60	0.59
Take butter	169	0.62	0.62	85	0.73	0.73	84	0.50	0.52
*Scores*									
Presence or absence of food groups score	164	0.50	0.57	80	0.60	0.69	84	0.42	0.48
Healthy unprocessed food score	164	0.57	0.79	80	0.67	0.88	84	0.48	0.72

Note: *n* = sample size; Rho = The Spearman correlation coefficient.

^a^ Not statistically significant agreement (Kappa) and/or not statistically significant correlation (Spearman correlation) (*p* > .05).

## Discussion

The current study examined the reliability of the SYBC-Q in a sample of Spanish children and adolescents. This addresses a key gap in the literature, as no previously reliable tools exist to specifically assess breakfast composition and quality in Spanish youth. The main findings suggest that the SYBC-Q showed an acceptable to good level of agreement and a moderate to high correlation between food groups, specific foods, and scores in the test–retest for children. In adolescents, the study found an acceptable to perfect level of agreement, but a low to high correlation between food groups, specific foods, and scores in the test–retest. Caution should be taken with some items of the questionnaire, specifically for children: the items ‘Take Cheese’ and ‘Take Milk Dessert’ in girls, and for adolescents, the items ‘Take Pâté’ in girls and ‘Take Dried Fruits’ in boys. These items showed a poor level of agreement and low correlation between the test and retest. Despite these limitations, the SYBC-Q represents a significant step forward as a reliable and easy tool for assessing breakfast composition and quality in Spanish children and adolescents.

Overall, the SYBC-Q demonstrated an acceptable to good level of agreement and a low to high correlation between test and retest across children, adolescents, and genders. Similar reliability ranges have been reported for short SFQs in Spanish children aged 7–9 years (Rho = 0.25–0.70) and in Italian school-aged children using the Breakfast Quality Score (kappa = 0.41–0.76) (Poinsot et al., 2024; Vioque et al., 2019). Our findings are also consistent with those reported by Moore et al. (2007), who assessed the reliability of an SFQ for breakfast composition in a sample of 374 Welsh children. That questionnaire showed an acceptable to moderate level of agreement between self-reported answers and interviews (kappa = 0.36–0.71), although it included only nine food items and was developed for a different cultural context. In contrast, the SYBC-Q includes 21 items and was specifically applied to Spanish children and adolescents. Our reliability coefficients are also aligned with more recent tools such as the KIDMED 2.0 (kappa = 0.40–0.78) and HBSC eating behaviours items (kappa = 0.33–0.79) used in European and Asian adolescent populations (Kohoutek et al., 2022; López-Gajardo et al., 2022). Nevertheless, there are still a limited number of brief and reliable tools designed to evaluate breakfast composition and quality (Adolphus et al., 2016). That review mainly included studies comparing breakfast consumption vs. skipping, with limited quality and little focus on breakfast composition. Golley et al. (2017) confirmed this gap, identifying few items that were both reliable and accurate, reinforcing the need for culturally specific tools like the SYBC-Q. The present results also align with those found in validated self-report instruments measuring frequency of food intake across the day (Notario-Barandiaran et al., 2020; Tayyem et al., 2021). For example, Notario-Barandiaran et al. (2020) reported correlations between 0.11 and 0.77 for a food frequency questionnaire in Spanish adolescents. Similarly, Tayyem et al. (2021) found correlations ranging from 0.08 to 0.95 in children and adolescents. These findings further support the reliability of the SYBC-Q as a useful and necessary instrument to monitor breakfast behaviours in young populations.

In addition, the SYBC-Q offers two methods for assessing breakfast quality, both of which demonstrated acceptable to moderate agreement and high test–retest correlations in children and adolescents. These results are consistent with other Spanish indices, including the Breakfast Quality Index trajectories described by Park et al. (2023) in early childhood and the INHES study in Italian youth (Martinez et al., 2024). These findings support the potential applicability and refinement of the Breakfast Quality Index, which has been the most widely used tool to assess breakfast quality in young Spanish populations (Arenaza et al., 2018; Cuadrado-Soto et al., 2020; Monteagudo et al., 2013). It consists of 4 items related to food groups but was not validated by Monteagudo et al. (2013). Therefore, the SYBC-Q is an interesting proposal, as it includes a larger number of items for assessing breakfast (21 vs. 4) and has proven to be reliable. In fact, its levels of agreement are consistent with those obtained by other self-reported tools for measuring dietary patterns, such as the KIDMED index, which showed a moderate degree of agreement (kappa = 0.59 (Rei et al., 2021) and 0.61(Štefan et al., 2017)). Therefore, the SYBC-Q represents a promising option for evaluating breakfast quality and identifying low-quality breakfast patterns in children and adolescents.

However, certain items showed a low level of agreement between test and retest. Among children, these were mainly related to dairy product consumption. In adolescents, low agreement was observed for the item ‘Take pâté’ among girls and ‘Take dried fruits’ among boys. Similar item-specific difficulties have been reported for dairy-related questions in short SFQs (Golley et al., 2017) and in Mediterranean diet screeners for youth (López-Gajardo et al., 2022). There are different hypotheses to explain these results. Moore et al. (2007) observed similar issues in their study, where the milk item had a kappa of 0.14. They suggested that children might indicate only one component of a mixed food (e.g., selecting ‘cereal’ but omitting ‘milk’ when both were consumed together), leading to underreporting. This aligns with the tendency of children to interpret questions literally, making it harder for them to distinguish between individual food components (McPherson et al., 2000). Family environment may also play a role in shaping young people's eating patterns (Aldridge et al., 2009). Furthermore, test–retest reliability is particularly challenging in younger populations due to developmental variability and shifting behaviours (de Vet et al., 2011). Moreover, literature suggests that children may struggle to complete questionnaires accurately (Pérez-Rodrigo et al., 2015), which may affect reliability. In adolescents, variability in responses may relate to less structured eating habits, greater independence from parents, and concerns about body image (Marshall et al., 2012). To improve the reliability of items with low agreement, clearer definitions and examples should be included. For instance, specifying forms of cheese (e.g., cheese spread), dried fruits (e.g., raisins or dates), or types of pâté (e.g., veal, chicken), and supporting these with images could enhance comprehension. Despite these isolated issues, it is important to highlight that at least 18 out of 21 items showed acceptable agreement, indicating that most children and adolescents understood and responded to the SYBC-Q appropriately.

A key strength of this study is that the SYBC-Q addresses an important gap in the assessment of breakfast composition and quality in Spanish children and adolescents. Developed by nutrition experts, the tool is reliable and suitable for use in both children and adolescents. Another strength lies in its ability to categorize foods as healthy or unhealthy, simplifying breakfast quality evaluation. The questionnaire includes 21 foods grouped into seven categories, allowing for a comprehensive assessment. However, some limitations should be acknowledged. The study was conducted in a single country, which may limit the generalizability of the findings to other contexts. The use of convenience sampling could also introduce response bias. Additionally, the study did not distinguish between behavioural variability (i.e., changes in dietary behaviour over time) and technical variability (i.e., inconsistencies due to the tool itself). The absence of a formal pilot test to assess the comprehensibility of the questionnaire may also be considered a limitation, although no issues were reported during administration, suggesting that the items were appropriate for the target age group. Moreover, the SYBC-Q evaluates breakfast quality primarily based on the presence of food groups and only includes portion size criteria in a limited number of items (e.g., sugar and honey). As such, a small quantity of a given food is often weighted equally to a larger portion, which may affect the accuracy of dietary quality assessment. Future adaptations could consider integrating more detailed portion size estimations to improve nutritional precision. Another limitation is that the SYBC-Q assesses breakfast intake only on weekdays, which may not fully capture eating habits throughout the week. Since breakfast habits may differ on weekends (Monteiro et al., 2017), future versions of the tool could include weekend-specific items to provide a more comprehensive overview of breakfast behaviours. Lastly, although the original study protocol included plans for validating the SYBC-Q, the absence of complementary dietary intake data (e.g., 24-h recalls or direct observation) prevented formal validation analyses. Therefore, the current findings reflect only the test–retest reliability of the tool. Future research should aim to establish the validity of the SYBC-Q through comparison with established dietary assessment methods and expert evaluation.

## Conclusion

The SYBC-Q is a reliable instrument for assessing breakfast consumption and quality in Spanish children and adolescents. However, specific items may need to be reviewed and possibly modified to enhance its reliability. Despite this, the SYBC-Q is simple and easy to administer, making it a valuable tool for healthcare professionals, researchers, and practitioners in monitoring breakfast composition and quality among this population. This tool will be instrumental in future studies analyzing the prevalence of breakfast nutritional patterns, as well as in research exploring the composition and quality of breakfast and their association with diseases (e.g., metabolic disorders) or other benefits (e.g., academic performance in school).

## Supplemental Material

sj-docx-1-nah-10.1177_02601060251368960 - Supplemental material for Measuring breakfast dietary patterns in Spanish youth: Reliability of the Spanish Youth Breakfast Consumption Questionnaire (SYBC-Q) Supplemental material, sj-docx-1-nah-10.1177_02601060251368960 for Measuring breakfast dietary patterns in Spanish youth: Reliability of the Spanish Youth Breakfast Consumption Questionnaire (SYBC-Q) by Pablo Campos-Garzón, Romina Gisele Saucedo-Araujo, Manuel Herrador-Colmenero and Yaira Barranco-Ruiz in Nutrition and Health

sj-docx-2-nah-10.1177_02601060251368960 - Supplemental material for Measuring breakfast dietary patterns in Spanish youth: Reliability of the Spanish Youth Breakfast Consumption Questionnaire (SYBC-Q) Supplemental material, sj-docx-2-nah-10.1177_02601060251368960 for Measuring breakfast dietary patterns in Spanish youth: Reliability of the Spanish Youth Breakfast Consumption Questionnaire (SYBC-Q) by Pablo Campos-Garzón, Romina Gisele Saucedo-Araujo, Manuel Herrador-Colmenero and Yaira Barranco-Ruiz in Nutrition and Health
