# Supplementary material for: Measuring breakfast dietary patterns in Spanish youth: Reliability of the Spanish Youth Breakfast Consumption Questionnaire (SYBC-Q)
Source: Nutr Health. 2025 Sep 1;32(4):1265–73. doi: 10.1177/02601060251368960 (PMC13365622; doi:10.1177/02601060251368960)
Supplement: sj-docx-2-nah-10.1177_02601060251368960 - Supplemental material for Measuring breakfast dietary patterns in Spanish youth: Reliability of the Spanish Youth Breakfast Consumption Questionnaire (SYBC-Q) [file sj-docx-2-nah-10.1177_02601060251368960.docx]

CUESTIONARIO DE ALUMNOS/AS

| **Fecha de nacimiento** | | | | | |
| --- | --- | --- | --- | --- | --- |
| **Día** | | **Mes** | | **Año** | |
| $$ | | $$ | | $$ | |
| 0U | 0U | 0U | 0U | 0U | 0U |
| 1U | 1U | 1U | 1U | 1U | 1U |
| 2U | 2U |  | 2U | 2U | 2U |
| 3U | 3U |  | 3U | 3U | 3U |
|  | 4U |  | 4U | 4U | 4U |
|  | 5U |  | 5U | 5U | 5U |
|  | 6U |  | 6U | 6U | 6U |
|  | 7U |  | 7U | 7U | 7U |
|  | 8U |  | 8U | 8U | 8U |
|  | 9U |  | 9U | 9U | 9U |

| **Nivel** | |
| --- | --- |
| 1UPrimaria | |
| 2USecundaria | |
| 3UBachillerato | |
| **Curso** | **Grupo** |
| U | U |
| 1U | AU |
| 2U | BU |
| 3U | CU |
| 4U | DU |
| 5U | EU |
| 6U | FU |

| **Eres** |
| --- |
| 1UNiño  2UNiña |

| **¿En qué país naciste …** | | |
| --- | --- | --- |
|  | España | Otro (escríbelo): |
| tú? | U | U |
| **¿En qué país nació …** | | |
|  | España | Otro (escríbelo): |
| tu Madre? | U | U |
| tu Padre? | U | U |


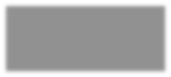

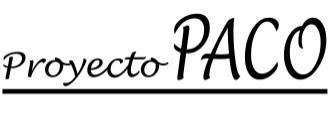


¡Hola! Nos gustaría que completaras este cuestionario. Ante todo, muchas gracias por tu colaboración. En todas las preguntas se debe de marcar solo una respuesta, a no ser que se indique lo contrario.


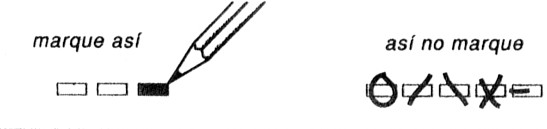


Es importante que **contestes a todas las preguntas** utilizando bolígrafo azul o negro, rellenando **completamente** los cuadros de la opción de respuesta elegida, tal y como se muestra en la imagen. Muchas gracias.

| **Fecha de hoy** | | | | | |
| --- | --- | --- | --- | --- | --- |
| **Día** | | **Mes** | | **Año** | |
| $$ | | $$ | | $$ | |
| 0U | 0U | 0U | 0U | 0U | 0U |
| 1U | 1U | 1U | 1U | 1U | 1U |
| 2U | 2U |  | 2U | 2U | 2U |
| 3U | 3U |  | 3U | 3U | 3U |
|  | 4U |  | 4U | 4U | 4U |
|  | 5U |  | 5U | 5U | 5U |
|  | 6U |  | 6U | 6U | 6U |
|  | 7U |  | 7U | 7U | 7U |
|  | 8U |  | 8U | 8U | 8U |
|  | 9U |  | 9U | 9U | 9U |

| **Hora de inicio (formato 24 horas)**  $$ : $$ | | | |
| --- | --- | --- | --- |
| 0U | 0U | 0U | 0U |
| 1U | 1U | 1U | 1U |
| 2U | 2U | 2U | 2U |
|  | 3U | 3U | 3U |
|  | 4U | 4U | 4U |
|  | 5U | 5U | 5U |
|  | 6U |  | 6U |
|  | 7U |  | 7U |
|  | 8U |  | 8U |
|  | 9U |  | 9U |

| **Centro** | |
| --- | --- |
| $$ | |
| 0U | 0U |
| 1U | 1U |
| 2U | 2U |
| 3U | 3U |
| 4U | 4U |
| 5U | 5U |
| 6U | 6U |
| 7U | 7U |
| 8U | 8U |
| 9U | 9U |

| **Código** | | | |
| --- | --- | --- | --- |
| $ | $ | $ | $ |
| 0U | 0U | 0U | 0U |
| 1U | 1U | 1U | 1U |
| 2U | 2U | 2U | 2U |
| 3U | 3U | 3U | 3U |
| 4U | 4U | 4U | 4U |
| 5U | 5U | 5U | 5U |
| 6U | 6U | 6U | 6U |
| 7U | 7U | 7U | 7U |
| 8U | 8U | 8U | 8U |
| 9U | 9U | 9U | 9U |

| **Cohorte** | |
| --- | --- |
| $$ | |
| 0U | 0U |
| 1U | 1U |
| 2U | 2U |
| 3U | 3U |
| 4U | 4U |
| 5U | 5U |
| 6U | 6U |
| 7U | 7U |
| 8U | 8U |
| 9U | 9U |

| **Edad** | |
| --- | --- |
| $$ | |
|  | 0U |
| 1U | 1U |
| 2U | 2U |
| 3U | 3U |
| 4U | 4U |
| 5U | 5U |
| 6U | 6U |
| 7U | 7U |
| 8U | 8U |
| 9U | 9U |

| **Nombres y Apellidos (MAYÚSCULA):** |
| --- |
| **Teléfono: Correo electrónico:** |
| **Dirección postal (MAYÚSCULA):**  (Calle, número, piso, letra) |
| **Ciudad/Pueblo: Código Postal:** |

**CENTRO EDUCATIVO SE REFIERE A TU COLEGIO O INSTITUTO**

1. **¿A qué distancia vives del centro educativo?**

| Menos de  0,5 km | De 0,5 a menos  de 1,5 km | De 1,5 a menos  de 3 km | De 3 a menos  de 6 km | 6 km o más |
| --- | --- | --- | --- | --- |
| U | U | U | U | U |

1. **¿Cuánto tardas en llegar al centro educativo desde que sales de tu casa?**

| Menos de 5  minutos | De 5´ a menos  de 15´ | De 15´ a menos  de 30´ | De 30´ a menos  de 60´ | 60 minutos  o más |
| --- | --- | --- | --- | --- |
| U | U | U | U | U |

1. **¿Cómo vas habitualmente al centro educativo? (Marca solo una opción. Si combinas varios modos de transporte, indica aquel en el que inviertes más tiempo)**

| Andando | Bici | Coche | Moto | Autobús  escolar | Autobús  público | Metro/  Tren/ Tranvía | Otros:  (escríbelo) |
| --- | --- | --- | --- | --- | --- | --- | --- |
| 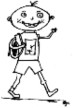 | 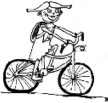 | 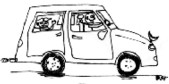 | 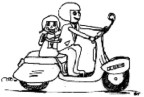 | 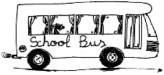 | 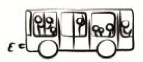 | 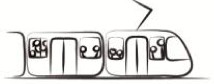 |  |
| U | U | U | U | U | U | U | U |

1. **¿Cómo vuelves habitualmente a casa? (Marca solo una opción. Si combinas varios modos de transporte, indica aquel en el que inviertes más tiempo)**

| Andando | Bici | Coche | Moto | Autobús  escolar | Autobús  público | Metro/  Tren/ Tranvía | Otros:  (escríbelo) |
| --- | --- | --- | --- | --- | --- | --- | --- |
| 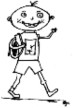 | 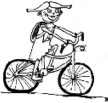 | 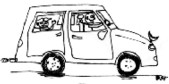 | 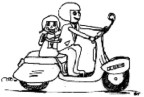 | 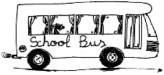 | 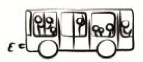 | 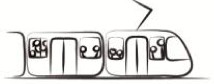 |  |
| U | U | U | U | U | U | U | U |

1. **¿Cuántos ordenadores (fijos o portátiles) tenéis en casa?**

| Ninguno | Uno | Dos | Más de dos |
| --- | --- | --- | --- |
| U | U | U | U |

1. **¿Tenéis algún vehículo motorizado de 4 ruedas (coche, furgoneta, camión o autocaravana) en casa?**

| Ninguno | Sí, uno | Sí, dos o más |
| --- | --- | --- |
| U | U | U |

1. **¿Tienes bicicleta en buenas condiciones para usar?**

Si U No U

**Piensa en los últimos 5 días que has tenido clase (sin incluir hoy) y contesta a las preguntas 8 y 9**

1. **¿Cómo FUISTE cada uno de los días al centro educativo? Puedes señalar más de una respuesta para cada día si has utilizado varios medios de transporte para ir al centro educativo.**

|  | **Lunes** | **Martes** | **Miércoles** | **Jueves** | **Viernes** |  |
| --- | --- | --- | --- | --- | --- | --- |
| **No fui al centro** | U | U | U | U | U |  |
| **Andando** | U | U | U | U | U | 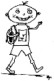 |
| **Bici** | U | U | U | U | U | 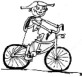 |
| **Coche** | U | U | U | U | U | 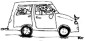 |
| **Moto** | U | U | U | U | U | 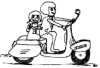 |
| **Autobús escolar** | U | U | U | U | U | 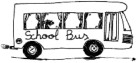 |
| **Autobús público** | U | U | U | U | U | 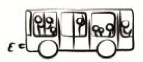 |
| **Metro/Tren/Tranvía** | U | U | U | U | U | 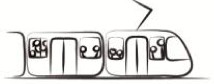 |
| **Otros:**  **(escríbelo)** | U | U | U | U | U |  |

1. **¿Cómo VOLVISTE cada uno de los días a casa? Puedes señalar más de una respuesta para cada día si has utilizado varios medios de transporte para volver desde el centro educativo.**

|  | **Lunes** | **Martes** | **Miércoles** | **Jueves** | **Viernes** |  |
| --- | --- | --- | --- | --- | --- | --- |
| **No fui al centro** | U | U | U | U | U |  |
| **Andando** | U | U | U | U | U | 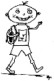 |
| **Bici** | U | U | U | U | U | 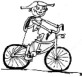 |
| **Coche** | U | U | U | U | U | 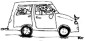 |
| **Moto** | U | U | U | U | U | 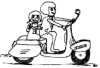 |
| **Autobús escolar** | U | U | U | U | U | 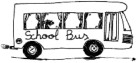 |
| **Autobús público** | U | U | U | U | U | 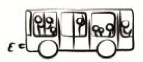 |
| **Metro/Tren/Tranvía** | U | U | U | U | U | 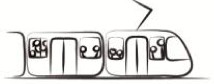 |
| **Otros:**  **(escríbelo)** | U | U | U | U | U |  |

1. **¿Qué opino sobre mi modo habitual de desplazarme al centro educativo?**

**Totalmente en Totalmente de desacuerdo (1) …… acuerdo (5)**

- 1. Mi modo habitual de ir al centro educativo se ajusta a lo que yo quiero

1 2 3 4 5

## U U U U U

1 2 3 4 5

- 1. Me siento capaz de ir andando o en bici al centro educativo………………. U U U U U
  2. Me siento muy cómodo/a cuando voy al centro educativo _1_ _2_ _3_ _4_ _5_

acompañado/a…………………………………………………………………..................... U U U U U

- 1. La forma de desplazarme al centro educativo coincide perfectamente 1 2 3 4 5

con como yo quiero ir………………………………………………………………………….. U U U U U

- 1. Tengo las habilidades necesarias para desplazarme andando o en bici al _1_ _2_ _3_ _4_ _5_

centro educativo sin problemas…………………………………………………………… U U U U U

- 1. Me relaciono de forma muy amistosa con los que me acompañan cuando 1 2 3 4 5

voy al centro educativo…………………………………………………………… U U U U U

1 2 3 4 5

- 1. La forma de desplazarme al centro educativo es la que me apetece……. U U U U U

1 2 3 4 5

- 1. Soy hábil para desplazarme al centro educativo andando o en bici……… U U U U U
  2. Siento que me puedo comunicar abiertamente con los que me 1 2 3 4 5

acompañan………………………………………………………………………………………….. U U U U U

- 1. Puedo elegir cómo desplazarme al centro educativo…………………………… 1 2 3 4 5

## U U U U U

- 1. Me siento capacitado para desplazarme en bici o andando al centro educativo...…………………………………………………………………………………………..

1 2 3 4 5

## U U U U U

- 1. Me siento muy cómodo/a con los/as que me acompañan…………………… 1 2 3 4 5

## U U U U U

**)**

| **11. Yo voy o iría al centro educativo andando o en bici… Nada verda**  **11.1.** Porque los demás me dicen que debo hacerlo…….…………………………….. | **dero (0**  0  U | **…Total**  1  U | **mente**  2  U | **verdade**  3  U | **ro (4)**  4  U |
| --- | --- | --- | --- | --- | --- |
| **11.2.** Porque me siento culpable cuando no lo hago…………………………………… | 0  U | 1  U | 2  U | 3  U | 4  U |
| **11.3.** Porque valoro los beneficios que tiene ir al centro educativo andando  o en bici……………………………………………………………………………………………… | 0  U | 1  U | 2  U | 3  U | 4  U |
| **11.4.** Porque creo que ir al centro educativo andando o en bici es divertido. | 0  U | 1  U | 2  U | 3  U | 4  U |
| **11.5.** Porque está de acuerdo con mi forma de ser…………………………………….. | 0  U | 1  U | 2  U | 3  U | 4  U |
| **11.6.** No sé para qué me sirve ir al centro educativo andando o en bici………. | 0  U | 1  U | 2  U | 3  U | 4  U |
| **11.7.** Porque mis amigos/familia/profesor me dicen que tengo que hacerlo. | 0  U | 1  U | 2  U | 3  U | 4  U |
| **11.8.** Porque me siento avergonzado si no lo hago …………………………………….. | 0  U | 1  U | 2  U | 3  U | 4  U |
| **11.9.** Porque para mí es importante ir al centro educativo andando o en bici habitualmente……………………………………………………………………………………. | 0  U | 1  U | 2  U | 3  U | 4  U |
| **11.10.** Porque considero que ir al centro andando o en bici forma parte de mí | 0  U | 1  U | 2  U | 3  U | 4  U |
| **11.11.** No veo por qué tengo que molestarme en ir al centro educativo andando o en bici……………………………………………………………………………….. | 0  U | 1  U | 2  U | 3  U | 4  U |
| **11.12.** Porque disfruto yendo al centro educativo andando o en bici.…………… | 0  U | 1  U | 2  U | 3  U | 4  U |
| - 1. Esta es una pregunta control, marca la opción 3…………………………………   2. Porque otras personas no estarán contentas conmigo si no voy al centro educativo andando o en bici……………………………………………………. | 0  U  0  U | 1  U  1  U | 2  U  2  U | 3  U  3  U | 4  U  4  U |
| **11.15.** No veo el sentido de ir al centro educativo andando o en bici……………. | 0  U | 1  U | 2  U | 3  U | 4  U |
| **11.16.** Porque veo que ir al centro educativo andando o en bici es como una parte fundamental de lo que soy……………………………………………………….. | 0  U | 1  U | 2  U | 3  U | 4  U |
| **11.17.** Porque siento que he fallado cuando no he ido un día al centro educativo andando o en bici………………………………………………………………. | 0  U | 1  U | 2  U | 3  U | 4  U |
| **11.18.** Porque pienso que es importante hacer el esfuerzo de ir al centro educativo andando o en bici regularmente………………………………………… | 0  U | 1  U | 2  U | 3  U | 4  U |
| **11.19.** Porque encuentro el ir al centro educativo andando o en bici una actividad agradable……………………………………………………………………………. | 0  U | 1  U | 2  U | 3  U | 4  U |
| **11.20.** Porque me siento bajo la presión de mis amigos/as familia para ir al centro educativo andando o en bici……………………………………………………. | 0  U | 1  U | 2  U | 3  U | 4  U |
| **11.21.** Porque considero que ir al centro educativo andando o en bici está de acuerdo con mis valores…………………………………………………………………….. | 0  U | 1  U | 2  U | 3  U | 4  U |
| **11.22.** Porque me pongo nervioso/a si no voy al centro educativo andando o en bici regularmente……….……….………………………………………………………… | 0  U | 1  U | 2  U | 3  U | 4  U |
| **11.23.** Porque me gusta el ir al centro educativo andando o en bici…………….. | 0  U | 1  U | 2  U | 3  U | 4  U |
| **11.24.** Pienso que ir al centro andando o en bici es una pérdida de tiempo…. | 0  U | 1  U | 2  U | 3  U | 4  U |

1. **¿Cuántos días a la semana tienes clase de Educación Física? (Marca solo una opción)**
   1. U 0 días (nunca)
   2. U 1 día
   3. U 2 días
   4. U 3 días
   5. U 4 días
   6. U 5 días (todos)
2. **¿Cuántos recreos tienes al día?**
   1. U 0 (ninguno)
   2. U 1
   3. U 2
   4. U 3
   5. U 4
3. **En general, ¿disfrutas haciendo actividad física?**
   1. U Nada
   2. U Poco
   3. U Algo
   4. U Bastante
   5. U Mucho
4. **¿Disfrutas en las clases de Educación Física en el centro educativo?**
   1. U Nada
   2. U Poco
   3. U Algo
   4. U Bastante
   5. U Mucho

A continuación, te preguntaremos sobre tu actividad física **en el centro educativo**. Esto incluye las clases de **Educación Física**, pero también la actividad que haces en los **recreos**, así como en el **camino** al **centro educativo** y en la **vuelta a casa**. Responde pensando en la actividad física que has hecho en el centro educativo durante **los últimos 7 días.**

1. **Ir al centro educativo: ¿Cuántos días fuiste andando o en bicicleta al centro educativo? (Marca solo una opción, si no lo recuerdas con exactitud, intenta señalar la respuesta más adecuada).**
   1. U 0 días (nunca)
   2. U 1 día
   3. U 2 días
   4. U 3 días
   5. U 4-5 días (todos los días)


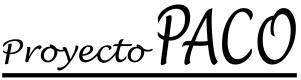


1. **Actividades durante las clases de Educación Física, ¿con qué frecuencia estuviste corriendo y moviéndote en juegos o actividades organizadas por el profesor/a? (si no tuviste Educación Física, elige “no tuve Educación Física”).**
   1. U No tuve Educación Física
   2. U Muy poco tiempo
   3. U Poco tiempo
   4. U Más o menos la mitad del tiempo
   5. U Mucho tiempo
   6. U Casi todo el tiempo
2. **Actividades durante los recreos: ¿Con qué frecuencia estuviste practicando deporte, andando, corriendo o jugando de forma activa? (si no tuviste recreos, elige “no tuve recreos en el centro”).**
   1. U No tuve recreos en el centro educativo
   2. U Muy poco tiempo
   3. U Poco tiempo
   4. U Más o menos la mitad del tiempo
   5. U Mucho tiempo
   6. U Casi todo el tiempo
3. **Actividad durante el descanso para comer: Durante el descanso para comer al mediodía en el comedor del centro educativo, ¿con qué frecuencia estuviste moviéndote, andando o jugando? (si no almorzaste en el comedor del centro, elige “no comí en el centro educativo”).**
   1. U No comí en el centro educativo
   2. U Muy poco tiempo
   3. U Poco tiempo
   4. U Más o menos la mitad del tiempo
   5. U Mucho tiempo
   6. U Casi todo el tiempo
4. **Volver del centro educativo: ¿Cuántos días volviste del centro andando o en bicicleta? (Marca solo una opción, si no lo recuerdas con exactitud, intenta señalar la respuesta más adecuada).**
   1. U 0 días (ningún día)
   2. U 1 día
   3. U 2 días
   4. U 3 días
   5. U 4-5 días (todos los días)

Estas preguntas son sobre tu nivel de **actividad física** en distintos periodos (**fuera del centro educativo**). Aquí se incluyen tanto las **actividades deportivas** estructuradas como **el** tiempo en el que juegas **con** amigos/as, bailas o haces **tareas de casa** (ordenar la habitación, limpiar, etc.). Responde pensando en la actividad física que has hecho fuera de centro educativo durante **los últimos 7 días.**

1. **Actividad antes de ir al centro educativo: Antes de ir al centro (entre las 6:00 y las 8:30- 9:00) ¿cuántos días hiciste actividad física durante al menos 10 minutos? (aquí se incluyen actividades realizadas en casa, pero NO otras como ir andando o en bicicleta al centro educativo) (Marca solo una opción).**
   1. U 0 días (ningún día)
   2. U 1 día
   3. U 2 días
   4. U 3 días
   5. U 4-5 días (todos los días)
2. **Actividad después de volver del centro educativo: Después de volver del centro (entre las 14:00- 14:30 y las 18:00), ¿cuántos días hiciste actividad física durante al menos 10 minutos? (se incluyen actividades como jugar con amigos/as y familia, deportes de equipo o clases en las que hagas actividad física, pero NO la vuelta del centro educativo andando o en bicicleta) (Marca solo una opción).**
   1. U 0 días (ningún día)
   2. U 1 día
   3. U 2 días
   4. U 3 días
   5. U 4-5 días (todos los días)
3. **Actividad por las tardes: Por las tardes (entre las 18:00 y las 22:00) ¿cuántos días hiciste actividad física durante al menos 10 minutos? (se incluyen actividades como jugar con amigos/as y familia, deportes de equipo o clases en las que hagas actividad física, pero NO la vuelta del centro educativo andando o en bicicleta) (Marca solo una opción).**
   1. U 0 días (ningún día)
   2. U 1 día
   3. U 2 días
   4. U 3 días
   5. U 4-5 días (todos los días)
4. **Actividad en sábados: ¿Cuánta actividad física hiciste el sábado pasado? (se incluye ejercicio físico, tareas de la casa, excursiones con la familia, deportes, baile o juegos. Si no lo recuerdas con exactitud, intenta señalar la respuesta más adecuada).**
   1. U Nada de actividad física (0 minutos)
   2. U Muy poca actividad física (1-30 minutos)
   3. U Una cantidad media de actividad física (31-59 minutos)
   4. U Mucha actividad física (1-2 horas)
   5. U Una gran cantidad de actividad física (más de dos horas)
5. **Actividad en domingos: ¿Cuánta actividad física hiciste el domingo pasado? (se incluye ejercicio, trabajos, excursiones con la familia, deportes, baile o juegos. Si no lo recuerdas con exactitud, intenta señalar la respuesta más adecuada).**
   1. U Nada de actividad física (0 minutos)
   2. U Muy poca actividad física (1-30 minutos)
   3. U Una cantidad media de actividad física (31-59 minutos)
   4. U Mucha actividad física (1-2 horas)
   5. U Una gran cantidad de actividad física (más de dos horas)

Estas preguntas son sobre el tiempo que pasas **descansando y sentado.** Probablemente estés sentado mientras **comes**, haces los **deberes** o tocas **instrumentos musicales**; pero también puedes estar sentado cuando ves la televisión, juegas a **videojuegos**, usas el **ordenador**, el **móvil**, **tablets/iPad** u otros. Responde a estas preguntas pensando en el tiempo que has pasado sin moverte durante estas actividades en **los últimos 7 días (solo en los días de clase).**

1. **Tiempo viendo televisión: ¿Cuánto tiempo estuviste viendo la televisión fuera del centro educativo? (incluye el tiempo que has estado viendo películas o deportes, pero NO jugando a videojuegos).**
   1. U No vi nada la televisión
   2. U Vi la televisión menos de 1 hora al día
   3. U Vi la televisión entre 1-2 horas al día
   4. U Vi la televisión más de 2 horas y hasta 3 horas al día
   5. U Vi la televisión más de 3 horas al día
2. **Tiempo con videojuegos: ¿Cuánto tiempo estuviste jugando a videojuegos fuera del centro educativo? (incluye jugar a la Nintendo DS, Wii, Xbox, PlayStation u otras consolas, NO incluir juegos con ordenador).**
   1. U No jugué nada con videojuegos
   2. U Jugué menos de 1 hora al día
   3. U Jugué entre 1-2 horas al día
   4. U Jugué más de 2 horas y hasta 3 horas al día
   5. U Jugué más de 3 horas al día
3. **Tiempo con ordenador: ¿Cuánto tiempo estuviste usando el ordenador fuera del centro educativo? (NO se incluye el uso para hacer deberes, pero SÍ en redes sociales como Facebook, navegando en internet, chateando, jugando a videojuegos o juegos online).**
   1. U No usé el ordenador para estas actividades
   2. U Usé el ordenador menos de 1 hora al día
   3. U Usé el ordenador entre 1-2 horas al día
   4. U Usé el ordenador más de 2 horas y hasta 3 horas al día
   5. U Usé el ordenador más de 3 horas al día
4. **Tiempo con teléfono móvil: ¿Cuánto tiempo estuviste usando tu móvil fuera del centro educativo? (Esto incluye el tiempo hablando por teléfono y escribiendo mensajes de texto o WhatsApp. Si no tienes móvil y tampoco usas nunca el de tus padres o algún amigo/a, elige la opción “no usé nunca el móvil”).**
   1. U No usé nunca el móvil
   2. U Usé el móvil menos de 1 hora al día
   3. U Usé el móvil entre 1-2 horas al día
   4. U Usé el móvil más de 2 horas y hasta 3 horas al día
   5. U Usé el móvil más de 3 horas al día
5. **Hábitos sedentarios en una semana normal (NO solo la última semana): ¿Cuál de las siguientes frases define mejor tus hábitos sedentarios en casa?**
   1. U Apenas estoy sentado en mi tiempo libre
   2. U Estoy sentado durante poco tiempo en mi tiempo libre
   3. U Estoy sentado una cantidad moderada de tiempo en mi tiempo libre
   4. U Estoy sentado mucho tiempo en mi tiempo libre
   5. U Estoy sentado casi todo el tiempo en mi tiempo libre
6. **De lunes a viernes durante las semanas que vas al centro educativo, ¿cuántos días desayunas habitualmente?**
   1. U Nunca desayuno en los días que voy al centro educativo
   2. U 1 día
   3. U 2 días
   4. U 3 días
   5. U 4 días
   6. U 5 días
7. **¿Cuál es la razón por la que habitualmente te saltas el desayuno?**
   1. U Nunca me salto el desayuno
   2. U No tengo tiempo suficiente
   3. U No me gusta la comida que hay en casa
   4. U Nunca me acuerdo
   5. U No tengo hambre por las mañanas
   6. U No puedo comer pronto por las mañanas
   7. U Otros:
8. **¿Qué tomas para desayunar habitualmente los días de la semana antes de ir al centro educativo? (Responde a todos los alimentos con sí o no).**

| **Alimentos** | **SI** | **NO** | **Alimentos** | **SI** | **NO** |
| --- | --- | --- | --- | --- | --- |
| - Leche | U | U | - Chocolate sin leche | U | U |
| - Yogurt natural | U | U | - Chocolate con leche, Cacao en polvo (Cola- cao, Nesquik, …) o nocilla/ nutella | U | U |
| - Queso | U | U | - Frutos secos | U | U |
| - Fruta natural en piezas (plátano, manzana, naranja, …) o zumo natural | U | U | - Aceite de oliva | U | U |
| - Zumo de frutas tetrabrik o fruta en almíbar | U | U | - Mantequilla o margarina | U | U |
| - Pan blanco (barra o molde, de avena, maíz, …) | U | U | - Postres lácteos (batidos de sabores, yogures o yogures para beber azucarados o grasos, natillas, flan, bebida de soja-avena) | U | U |
| - Pan integral (barra o molde, de avena, maíz, …) | U | U | - Bollería (galletas María, Tosta rica, Príncipe, Oreo, …. Magdalenas, sobaos, croissants, napolitanas, …) | U | U |
| - Cereales sin azúcares añadidos o Copos de avena | U | U | - Jamón serrano, jamón cocido, fiambre de pollo/pechuga de pavo, … | U | U |
| - Cereales de desayuno   (chocapic, Nesquik, Kellogs, …) | U | U | - Huevos (fritos, revueltos, cocidos, tortilla...) | U | U |
| - Azúcar (más de 1 cucharada) | U | U | - Patés, foie gras, otros fiambres grasos (mortadela, salami, chorizo, salchichón, fuet) | U | U |
| - Miel (hasta una cucharada) | U | U | - Tomate, aguacate, zanahoria, … | U | U |
| - Mermelada, confitura,.. | U | U |  |  |  |

- 1. **¿A qué hora te duermes habitualmente los días antes de clase?**
  2. **¿A qué hora te despiertas habitualmente los días de clase?**

| **Hora de dormirse**  $$ : $$ | | | |
| --- | --- | --- | --- |
| 0U | 0U | 0U | 0U |
| 1U | 1U | 1U | 1U |
| 2U | 2U | 2U | 2U |
|  | 3U | 3U | 3U |
|  | 4U | 4U | 4U |
|  | 5U | 5U | 5U |
|  | 6U |  | 6U |
|  | 7U |  | 7U |
|  | 8U |  | 8U |
|  | 9U |  | 9U |

Formato 24 horas

| **Hora de despertarse**  $$ : $$ | | |
| --- | --- | --- |
| 0U 0U | 0U | 0U |
| 1U 1U | 1U | 1U |
| 2U 2U | 2U | 2U |
| 3U | 3U | 3U |
| 4U | 4U | 4U |
| 5U | 5U | 5U |
| 6U |  | 6U |
| 7U |  | 7U |
| 8U |  | 8U |
| 9U |  | 9U |

35. ¿Es agradable la zona donde vives para salir a pasear o ir en bicicleta?

| **La zona donde vivo:** | Muy en  desacuerdo | Algo en  desacuerdo | Algo de  acuerdo | Muy de  acuerdo |
| --- | --- | --- | --- | --- |
| 1 Ofrece un ambiente agradable para  caminar e ir en bicicleta | U | U | U | U |
| **En la zona donde vivo:** | Ninguno | Unos pocos | Bastantes | Muchos |
| 2 Los edificios están limpios y sin pintadas (grafiti) | U | U | U | U |
| 3 Hay bastante árboles en las calles | U | U | U | U |
| 4 Hay edificios mal mantenidos, vacíos o feos | U | U | U | U |

| **36. Para mí es difícil ir andando o en bici al centro educativo porque:** | | **Totalmente en desacuerdo (1) ……** | | **Totalmente de acuerdo (4)** | |
| --- | --- | --- | --- | --- | --- |
| a. No hay aceras ni carriles bici……………………………….……………………. | | 1  U | 2  U | 3  U | 4  U |
| b. | El camino es aburrido……………………………………………………….......... | 1  U | 2  U | 3  U | 4  U |
| c. El camino no tiene una buena iluminación………………………………… | | 1  U | 2  U | 3  U | 4  U |
| d. Hay uno o más cruces peligrosos………………………………………………. | | 1  U | 2  U | 3  U | 4  U |
| e. Paso demasiado calor y sudo, o llueve siempre………………………… | | 1  U | 2  U | 3  U | 4  U |
| f. Otros niños/as no van andando o en bicicleta...……………………….. | | 1  U | 2  U | 3  U | 4  U |
| g. No se considera guay ir andando o en bicicleta………………………… | | 1  U | 2  U | 3  U | 4  U |
| h. Voy demasiado cargado con cosas…………….……………………………… | | 1  U | 2  U | 3  U | 4  U |
| i. Es más fácil ir conduciendo o que me lleven…………………………….. | | 1  U | 2  U | 3  U | 4  U |
| j. Es necesaria demasiada planificación previa…………………………….. | | 1  U | 2  U | 3  U | 4  U |
| k. No hay sitios donde dejar la bicicleta con seguridad…………………. | | 1  U | 2  U | 3  U | 4  U |
| l. | Hay perros callejeros…………………………………………...…………………… | 1  U | 2  U | 3  U | 4  U |
| m. | Está muy lejos…………………………………………………………………………… | 1  U | 2  U | 3  U | 4  U |
| n. Tendría que caminar/ir en bicicleta por lugares que serían inseguros debido a la delincuencia u otras cosas relacionadas con la delincuencia (por ejemplo, el vandalismo, los grafitis, gente bebiendo alcohol en lugares públicos)………………………………………. | | 1  U | 2  U | 3  U | 4  U |
| o. No disfruto yendo andando o en bicicleta al centro educativo…. | | 1  U | 2  U | 3  U | 4  U |
| p. | Hay demasiadas cuestas……………………………………………………………. | 1  U | 2  U | 3  U | 4  U |
| q. | Hay demasiado tráfico…………………………………..………………………….. | 1  U | 2  U | 3  U | 4  U |
| r. Los carriles-bici están ocupados por personas que van andando.. | | 1  U | 2  U | 3  U | 4  U |

37.1. ¿Con quién vas al centro educativo? 37.2. ¿Con quién vuelves del centro educativo? (Puedes marcar varias opciones)

| a. Yo solo o sola……………………….…. | U | a. Yo solo o sola ……………….………… | U |
| --- | --- | --- | --- |
| b. Con mi madre………………............. | U | b. Con mi madre ……………….……….. | U |
| c. Con mi padre………………............ | U | c. Con mi padre.……….................. | U |
| d. Con mi abuela………………........... | U | d. Con mi abuela……………………….. | U |
| e. Con mi abuelo………………........... | U | e. Con mi abuelo………………........... | U |
| f. Con otros niños/adolescentes… | U | f. Con otros niños/adolescentes… | U |
| g. Con otros adultos………….……….. | U | g. Con otros adultos………….……….. | U |
| **38. Si vas acompañado/a habitualmente de un adulto (padre, madre, abuelo, etc.) explica por qué respondiendo a todas las preguntas con sí o no. Si vas solo o con otros jóvenes, pasa a la pregunta 39.** | | | |
| **SI NO**   1. Vivo lejos del centro educativo………………………………………………………… U U 2. Mis padres no me dejan ir solo/a…………………………………………………….. U U 3. No me gusta ir solo/a………………………………………………………………………. U U 4. Hay mucho tráfico…………………………………………………………………………… U U 3. Llevo mucho peso en mi mochila…………………………………………………….. U U 4. A mis padres les viene bien porque me dejan de camino al trabajo…. U U 5. A mis padres les viene bien porque tengo hermanos/as pequeños/as U U 6. Otros: U U | | | |

1. **Si pudieras elegir:**
   1. **¿Cómo te gustaría ir al centro educativo? 39.2. ¿Cómo te gustaría volver del centro?**

(Marca solo una opción)

| 1. Andando.....................……. | U | 1. Andando……………............... | .U |
| --- | --- | --- | --- |
| 2. Bicicleta…………………………. | U | 2. Bicicleta…………………………… | U |
| 3. Coche…………………………….. | U | 3. Coche………………………………. | U |
| 4. Moto……………………………… | U | 4. Moto……………………………….. | U |
| 5. Autobús escolar…………..… | U | 5. Autobús escolar…………….... | U |
| 6. Autobús público…………….. | U | 6. Autobús público………………. | U |
| 7. Metro/tren/tranvía…..…... | U | 7. Metro/tren/tranvía……….... | U |
| 8. Patinete………………………….  9. Otros: | U  U | 8. Patinete……………………………  9. Otros: | U  U |

#
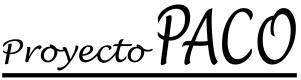
 

**RECUERDA QUE ES MUY IMPORTANTE HABER RESPONDIDO A TODAS LAS PREGUNTAS Y NO DEJAR NINGUNA EN BLANCO.**

**¡COMPRUEBA QUE TODO ESTÁ RELLENO POR FAVOR!**

1. **¿Tienes alguna sugerencia/observación? ¡Cuéntanosla!**

| **Hora de fin (formato 24h)**  $$ : $$ | | | |
| --- | --- | --- | --- |
| 0U | 0U | 0U | 0U |
| 1U | 1U | 1U | 1U |
| 2U | 2U | 2U | 2U |
|  | 3U | 3U | 3U |
|  | 4U | 4U | 4U |
|  | 5U | 5U | 5U |
|  | 6U |  | 6U |
|  | 7U |  | 7U |
|  | 8U |  | 8U |
|  | 9U |  | 9U |

¡ENHORABUENA, HAS TERMINADO!

**GRACIAS POR TU COLABORACIÓN**
